# Effective use of *yokukansan* for Caucasian patient with panic disorder: A case report

**DOI:** 10.1002/pcn5.231

**Published:** 2024-07-29

**Authors:** Noriaki Ohsako, Hiroshi Kimura

**Affiliations:** ^1^ Department of Psychiatry Bellvitge University Hospital‐IDIBELL Barcelona Spain; ^2^ Department of Psychiatry Chiba University Graduate School of Medicine Chiba Japan; ^3^ Department of Psychiatry Gakuji‐kai Kimura Hospital Chiba Japan; ^4^ Department of Psychiatry International University of Health and Welfare Narita Chiba Japan

**Keywords:** 5‐HT receptor, ethnicity, herbal medicine, panic disorders, *yokukansan*

## Abstract

**Background:**

*Yokukansan* is a traditional Japanese herbal medicine that is widely administered to individuals of various age groups as an effective drug for anxiety, with few side‐effects. While animal studies have yielded promising results concerning *yokukansan*'s potential in treating anxiety disorders, comprehensive validation has remained incomplete. Moreover, most of the clinical investigations regarding *yokukansan* have primarily focused on Japanese subjects, and its impact on non‐Asian ethnicities remains unverified.

**Case Presentation:**

We present the case of a 17‐year‐old Caucasian female with panic disorder (PD). Following her relocation to Japan, she experienced panic attacks due to environmental changes, which subsequently prompted her to visit to our clinic. Various medical examinations revealed no abnormalities, which ruled out the possibility of any physical illness other than PD. Significantly, the administration of *yokukansan* resulted in a notable reduction in panic attacks, as well as in anticipatory anxiety, accompanied by discernible enhancements in psychosocial functioning and overall quality of life. Furthermore, it is imperative to underscore the fact that no noteworthy adverse events took place.

**Conclusion:**

Panic attacks and profound anxiety in a Caucasian patient with PD were successfully treated with the use of *yokukansan*. This case study suggests that *yokukansan* may be effective in treating PD in Caucasians as well as in Asians. However, to substantiate this preliminary observation, further investigations are required.

## Graphical abstract

This report elucidates the effects of *yokukansan*, a traditional Japanese herbal medicine, on a typical panic attack in a 17‐year‐old Caucasian female. She experienced panic attacks approximately three times a week due to environmental changes, which subsequently prompted her to visit to our clinic, and the administration of *yokukansan* resulted in a notable reduction in panic attacks, as well as in anticipatory anxiety, accompanied by discernible enhancements in psychosocial functioning and overall quality of life. This case is noteworthy for the fact that the panic attacks and profound anxiety in a non‐Asian patient with panic disorder were successfully treated with the use of *yokukansan*, with no adverse events observed.

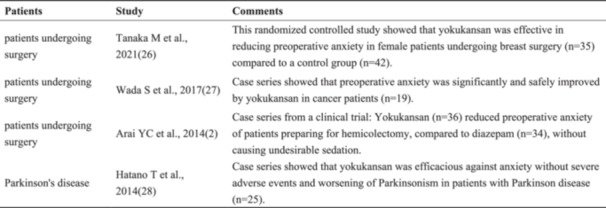

## BACKGROUND

*Yokukansan*, a traditional herbal remedy originating in Japan (Kampo), earned official approval within Japan for its therapeutic utility for addressing neurosis, insomnia, and night‐crying and irritability in pediatric populations. In recent years, a growing body of research has unveiled its efficacy in treating behavioral and psychological symptoms of dementia.^1^ It consequently gained widespread utilization as a pharmaceutical agent suitable for individuals of diverse gender orientations and age cohorts, characterized by minimal side‐effects.^2^

Noteworthy findings obtained from in vitro experimentation and murine investigations elucidated the capacity of *yokukansan* to stimulate the 5‐HT1A receptor^3^, ^4^, ^5^, ^6^ and to downregulate the 5‐HT2A receptor.^7^, ^8^ These actions collectively suggest a trend toward demonstrating antidepressant and anxiolytic effects of *yokukansan*. While the suggested therapeutic effects of Kampo medicines concerning anxiety disorders appear promising in many studies,^9^ they are yet to attain exclusive validation as there are still many unknowns regarding their pharmacological effects. Furthermore, most clinical studies conducted on Asians, as well as the effects on other racial groups, such as Caucasians and individuals of African descent, have yet to be confirmed.

## CASE PRESENTATION

A 17‐year‐old Peruvian Caucasian female (herein referred to as Patient X) presented to the emergency department at night with a chief complaint of accelerated breathing, a feeling of dyspnea, palpitations, and headache.

At the time of her visit, there were frequent small to medium earthquakes in Japan, which often triggered a sense of disquietude in X. Preceding her hospital visit, similar symptoms had appeared during an encounter with a peer, occurring approximately three times weekly without any discernible provocation. On the day of the visit, a moderate‐scale earthquake had occurred, exacerbating her unease and precipitating symptoms that included heightened respiration, pronounced anxiety, and generalized tremors. These symptoms persisted unabated, prompting her to seek medical consultation, accompanied by her family.

X was born and raised in Peru, where she attended high school. Approximately three months preceding her initial visit, she had relocated to Japan, where her divorced mother resided, in response to the deteriorating COVID‐19 situation in Peru. She had continued her high school education via online classes conducted during the late evening hours, from 10:00 p.m. to 4:00 a.m. Japan Standard Time. Although she was a native Spanish speaker and was proficient in English, she had limited Japanese language skills. Consequently, despite her sociable disposition, her social interactions in Japan were primarily confined to her family. She lived with her mother and sister, with whom she had a good relationship. There was no significant medical or family history related to neurological or psychiatric disorders.

At her initial evaluation, her vital signs were normal and physical examination revealed no neurological abnormalities. A head CT scan and electrocardiogram showed no abnormal findings of note, and blood tests revealed no abnormalities in terms of thyroid, renal and liver function, electrolytes, complete blood cell count, or blood glucose levels. X was cleanly dressed, conversed fluently with the examiner in English and Spanish, maintained eye contact, and communicated calmly and expressively. Both she and her family denied any history of tobacco or alcohol use, as well as any consumption of illicit substances. Additionally, her symptoms were not related to problems with her menstrual cycle.

X reported experiencing autonomic manifestations, including palpitations, diaphoresis, diffuse tremulousness, and dyspnea, in addition to physical symptoms, such as thoracic discomfort and vertigo. She further described experiencing an altered sense of reality during these episodes, coupled with an overpowering apprehension of impending mortality. Symptoms indicative of depression, such as depressed mood and suicidal ideation, were absent. She was reassured by the fact that she had no problems with verbal interactions with her examiner, and talked about the burden of her online evening classes and her continued anxiety due to her linguistic and social isolation due to being in Japan. She also shared her concerns that, although talking to her friends in Peru relieved her anxiety, the time difference did not make interaction easy. Consequently, X was diagnosed with *DSM‐5* ^10^ panic disorder (PD), which was triggered by the change in environment away from her home country and the stress of taking classes at night. At the time of the initial visit, her Panic Disorder Severity Scale (PDSS)^11^ score was 10 and her Clinical Global Impressions‐Severity of illness (CGI‐S)^12^ was 4.

She was prescribed 2.5–5 g of *yokukansan* per day so that she could use it when panic attacks appeared or were about to happen. Because of the lack of immediate improvement in her living circumstances and the difficulties associated with air travel due to panic attacks, she persisted with her residence in Japan and experienced panic attacks approximately three times weekly. Over time, due to the effective use of *yokukansan*, the frequency of these attacks gradually waned, ultimately nearly disappearing completely after 5 weeks. Subsequently, X, in consultation with her family, made the decision to return to Peru, which served to further assuage her symptoms. Although she continued to use *yokukansan*, panic attacks rarely appeared anymore. At her final follow‐up, 8 months after her initial visit, her symptoms had markedly improved, with a PDSS score of 4 and a CGI‐S of 1.

## DISCUSSION

This case report exemplifies the utilization of *yokukansan* in a patient experiencing persistent typical panic attack manifestations, encompassing autonomic symptoms like palpitations and diaphoresis, as well as profound anxiety, such as fear. It is noteworthy that the use of *yokukansan* was not only effective in reducing panic attacks and anticipatory anxiety but also demonstrated notable enhancements in psychosocial functionality and quality of life—paramount objectives in the treatment of PDs.^13^ In our patient's case, comprehensive assessments, including a head CT scan, electrocardiogram, blood tests, and physical examination, effectively ruled out alternative physical pathologies aside from PD. To the best of our knowledge, this is the first report to describe the effects of *yokukansan* on PD. Only a few previous studies have examined the efficacy of *yokukansan* for anxiety disorders, an umbrella concept within *DSM‐5* ^10^ that includes PD. Animal studies demonstrated the anxiolytic effects of *yokukansan* via 5‐HT1A and 5‐HT2A receptors,^6^, ^8^ suggesting *yokukansan* as a prospective therapeutic modality for anxiety disorders and a variety of psychiatric disorders that co‐occur with anxiety.

The potential anxiolytic effects of *yokukansan* in human subjects were evidenced by its impact on anxiety symptoms related to physical illness (see Table 1). These investigations revealed that *yokukansan* exhibits favorable tolerability and anxiolytic properties. Conversely, the potential anxiolytic effects of *yokukansan* in human subjects with PD or anxiety disorders have not yet been conclusively demonstrated. Various validation studies of *yokukansan* in human cohorts have been conducted with respect to the behavioral and psychological symptoms of dementia, highlighting its effects on agitation, aggression, and other psychiatric symptoms, along with its favorable tolerability profile.^17^ In contrast, one study on postoperative delirium reported no effect on anxiety,^18^ and in this regard *yokukansan*'s pharmacological effects remain largely unknown. Nevertheless, the modest yet discernible efficacy and favorable tolerability of *yokukansan* for anxiety have been well demonstrated at the clinical level, and it is used routinely, especially in Japanese clinical settings. In this case, the anxiety symptoms were accompanied by physical symptoms, as well as a severe agitation, so we opted to administer *yokukansan*, which was effective in improving the symptoms.

**Table 1:** *Yokukansan* was reported to be potentially effective for patients with anxiety.

Patients	Study	Comments
Patients undergoing surgery	Tanaka et al.^14^	This randomized controlled study showed that *yokukansan* was effective in reducing preoperative anxiety in female patients undergoing breast surgery (*n* = 35) compared to a control group (*n* = 42).
Patients undergoing surgery	Wada et al.^15^	This case series showed that preoperative anxiety was significantly and safely improved by *yokukansan* in cancer patients (*n* = 19).
Patients undergoing surgery	Arai et al.^2^	In this case series from a clinical trial, *yokukansan* (*n* = 36) effectively reduced preoperative anxiety of patients preparing for hemicolectomy, compared to diazepam (*n* = 34), without causing undesirable sedation.
Parkinson's disease	Hatano et al.^16^	This case series showed that *yokukansan* was efficacious against anxiety without severe adverse events and worsening of Parkinsonism in patients with Parkinson's disease (*n* = 25).

All of these studies were limited to clinical settings in Asia, and particularly in Japan, and did not adequately examine racial differences. Significant racial differences among proposed biological markers for various psychiatric disorders indicated the importance of considering ethnic and racial factors in psychiatric research.^19^ Although there have since been many analyses to determine the effects of pharmacotherapy, their validation vis‐à‐vis racial and ethnic diversity remains questionable. In this context, the present case report, wherein the efficacy of *yokukansan* manifested itself in a non‐Asian patient, holds clinical significance and underscores its potential applicability in this regard.

As a general practice, PD is managed by a combination of pharmacological interventions and psychotherapeutic modalities. Foremost among pharmacological options are selective serotonin reuptake inhibitors (SSRIs), categorized as antidepressants,^20^ alongside short‐term usage of benzodiazepines.^21^ However, antidepressants are associated with gastrointestinal side‐effects, such as nausea and diarrhea,^22^ as well as a possible increased risk of suicidal behavior or thoughts,^23^ and benzodiazepines are linked to cognitive dysfunction or dependence,^24^, ^25^ which may present difficulties with tolerability. In the context of the present case, avoidance of antidepressants and benzodiazepines stemmed from concerns regarding tolerability issues. Conversely, *yokukansan* presents a more favorable tolerability profile, with preliminary indications suggesting its potential to improve psychiatric symptoms, including anxiety, and be devoid of severe adverse events,^17^ although large‐scale validation is still lacking. Pseudoaldosteronism has been reported as a serious adverse event linked to glycyrrhizin, which is contained in many Kampo medicines, including *yokukansan*,^26^ with its occurrence being dose‐dependent.^27^ Even though the medical package insert prescribes a daily dosage of 7.5 g of *yokukansan*,^28^ the patient was very anxious about the medications, so we initiated the treatment with a lower dose. The relatively lower dosage range of 2.5–5 g administered to this patient could potentially account for the absence of side‐effects.

The effectiveness of Kampo medicines for PD was reported for medications other than *yokukansan*. In a previous study, the effects on PD appeared 2–12 weeks after administration of the medicines.^9^ In this case, PD symptoms improved 5 weeks after administration of *yokukansan*, which was continued for 8 months. The time until symptom improvement was similar between previous reports and this case. Larger‐scale verification is also needed on this point.

Limitations intrinsic to this study encompass the absence of toxicology screening (which was not performed because supporting medical history from the patient and her family members suggested no drinking or drug abuse), the possibility that the examiner's English or Spanish language ability and the psychoeducation provided by the examiner were effective in treating PD and the inherent nature of this study as a case report.

## CONCLUSION

Panic attacks and profound anxiety in a Caucasian patient with PD were successfully treated with *yokukansan*. This case study suggests that *yokukansan* may be effective in treating PD and that it may be effective for Caucasians as well as for Asians. However, to substantiate this initial observation, further investigative inquiry is needed.

## AUTHOR CONTRIBUTIONS

Noriaki Ohsako acquired case data. Noriaki Ohsako and Hiroshi Kimura drafted and revised the manuscript. All authors read and approved the final manuscript.

## CONFLICT OF INTEREST STATEMENT

The authors declare no conflict of interest.

## ETHICS APPROVAL STATEMENT

This study was conducted according to the principles of the Declaration of Helsinki.

## PATIENT CONSENT STATEMENT

Written informed consent for presentation of the patient's clinical course was given by the patient.

## CLINICAL TRIAL REGISTRATION

Not applicable.

## Data Availability

Not applicable.
